# Process monitoring using inflated beta regression control chart

**DOI:** 10.1371/journal.pone.0236756

**Published:** 2020-07-30

**Authors:** Luiz M. A. Lima-Filho, Tarciana Liberal Pereira, Tatiene C. Souza, Fábio M. Bayer

**Affiliations:** 1 Departamento de Estatística, Universidade Federal da Paraíba, João Pessoa, Brazil; 2 Departamento de Estatística and LACESM, Universidade Federal de Santa Maria, Santa Maria, Brazil; Tongii University, CHINA

## Abstract

This paper provides a general framework for controlling quality characteristics related to control variables and limited to the intervals (0, 1], [0, 1), or [0, 1]. The proposed control chart is based on the inflated beta regression model considering a reparametrization of the inflated beta distribution indexed by the response mean, which is useful for modeling fractions and proportions. The contribution of the paper is twofold. First, we extend the inflated beta regression model by allowing a regression structure for the precision parameter. We also present closed-form expressions for the score vector and Fisher’s information matrix. Second, based on the proposed regression model, we introduce a new model-based control chart. The control limits are obtained considering the estimates of the inflated beta regression model parameters. We conduct a Monte Carlo simulation study to evaluate the performance of the proposed regression model estimators, and the performance of the proposed control chart is evaluated in terms of run length distribution. Finally, we present and discuss an empirical application to show the applicability of the proposed regression control chart.

## 1 Introduction

Standard control charts are directly applied to the output of a quality characteristic. However, the quality characteristic (process output) can be affected by external covariates (control variables), where we rather control a varying mean than a constant one. In these cases, the regression control chart [1] may be an effective statistical process control tool. Such method is widely used when the quality of a process or product is better characterized by a functional relationship between the response variable and one or more explanatory variables [2].

The standard regression control chart is based on the linear regression model, where the variable of interest is assumed to be normally distributed. However, in practice, several of these variables may not follow a normal distribution, leading to poor Gaussian-based inferences. Thus, several studies have been proposing non-Gaussian model-based control charts. [3] presented a model-based scheme for monitoring multiple gamma-distributed variables. By considering that robust methods can be effective in the presence of outlying observations, [4] explored the robust generalized linear model for a gamma-distributed response. [5] used deviance residual for monitoring variables in a three-stage process assuming gamma, normal, and Poisson distributions.

Examples of a non-Gaussian process output are variables that assume values in the standard unit interval, such as fractions and proportions. In such instances, the usual regression control chart may be inappropriate since double bounded data are typically asymmetric and the Gaussian-based assumption is not suitable. In this sense, [6] proposed the beta regression control chart to monitor fractions and proportions related to control variables. The proposed control chart considers the beta regression model with varying dispersion [7], assuming that the mean and dispersion parameters of beta distributed variables are related to exogenous variables and modeled by regression structures. However, fractions and proportions may contain zeros and/or ones, leading to the unsuitable use of the beta distribution for data modeling [8].

Alternative regression models have been proposed to mend beta regression flaws in the presence of zeros and/or ones. [9] presented a unit inflated beta model for modeling efficiency scores as a function of exogenous variables. [10] proposed a zero inflated beta model to analyze data in corporate capital structures. [8] introduced a general class of zero or one inflated beta regression models, which is a natural extension of the beta regression model [11] to model variables that assume values in (0, 1] or [0, 1). [12] proposed an inflated beta regression model based on a reparametrization of the inflated beta distribution. This model accommodates mixed random variable responses, with non-negligible probabilities of assuming zeros and/or ones and continuous values in the interval (0, 1) that follows a beta distribution. The inflated beta regression model introduced by [12] may be useful for developing model-based control charts for monitoring inflated beta distributed processes as it considers an interesting parametrization in terms of the response variable mean. However, the model proposed by [12] does not consider a regression structure for the precision parameter. The monitoring of the mean and precision (or dispersion) is relevant to the statistical process control [6, 13, 14]. In addition, incorrect modeling of the dispersion can generate a high number of false alarms or loss of detection power of special causes [6]. Moreover, dispersion modeling is necessary in regression models in order to obtain accurate inferences about the structure parameters of the mean regression [15].

Control chart is a dynamic tool that works under two different phases, namely Phase I and Phase II. In practical situations, the in-control parameters are unknown and have to be estimated from a Phase I data set. Different Phase I data sets lead to different control chart performance. Thus, it is important to study the practitioner-to-practitioner variability due to parameter estimation. The aim of Phase I analysis is to estimate the parameters, while quick detection of out-of-control state is conducted in Phase II [16]. The literature offers some studies related to Phase I and Phase II analyses in regression models. For example, [17] proposed Phase I profile monitoring schemes for binary responses that can be represented by logistic regression models. [17] developed several Hotelling *T*^2^-type Phase I control charts for monitoring the parameters of a logistic regression linking to a binary response and one or more predictor variables. [18] developed control charts by integrating an exponentially weighted moving average scheme with a likelihood ratio test based on logistic regression models in Phase II study. [19] proposed a new modeling and monitoring framework for Phase I analysis of multivariate profiles by incorporating the regression-adjustment technique into the functional principal components analysis. [20] proposed the monitoring of profiles using generalized linear models during Phase II in which the explanatory variables can be a fixed design or any random arbitrary design.

In this context, this paper introduces the inflated beta regression control chart (IBRCC) with varying dispersion, useful for monitoring double bounded variables when zeros or ones appear in the data along with the presence of control variables. The process output may represent individual measures (e.g. efficiency score) or a ratio between continuous numbers (e.g. relative humidity). The contribution of the present paper is twofold. First, we extend the inflated beta regression model proposed by [12] by allowing a regression structure for the precision parameter. We also discuss likelihood inference of the model parameters. Second, we introduce the IBRCC based on the proposed inflated beta regression model with varying dispersion. Since in practice the parameters of the regression model are unknown, the proposed control chart is implemented into two phases. In Phase I, the parameters are estimated from an in-control sample, and in Phase II, we perform the monitoring scheme.

The remaining of the paper unfolds as follows. In Section 2, we describe the IBRCC and introduce the beta inflated mean regression model with varying dispersion. We also discuss likelihood inference and present the control limits estimation procedure. Section 3 presents a simulation study to evaluate (i) the inflated beta regression model with varying dispersion estimators and (ii) the performance of the proposed IBRCC and some competing control charts in the literature based on the run length (RL). In Section 4, we discuss and present an empirical application to show the applicability of the proposed IBRCC in real situations. Finally, some conclusions are presented in Section 5.

## 2 Inflated beta regression control chart

In this section, we introduce the IBRCC. Firstly, in Subsection 2.1 we present the inflated beta regression model with varying dispersion. The model we propose in this work is an extension of the model proposed by [12], where the authors used a reparametrization of the inflated beta distribution indexed by the response mean. In the Subsection 2.2 we present the model-based control limits for the proposed IBRCC. Secondly, in Subsubsection 2.2.1, we discuss the likelihood inference for the model parameters. Finally, in Subsubsection 2.2.2 we present the control limits estimation procedure.

### 2.1 Inflated beta regression model with varying dispersion

The inflated beta density function is given by [12]

$$\begin{array}{c} f\left(y;\alpha_{0},\alpha_{1},\gamma ,\phi \right)=\{\begin{array}{ccc} \alpha_{0}\left(1-\gamma \right), & \text{if} & y=0,\\ cB(y;\mu ,\phi ), & \text{if} & y\in (0,1),\\ \alpha_{1}\gamma , & \text{if} & y=1, \end{array} \end{array}$$ (1)

where 0 < *α*_0_ < 1, 0 < *α*_1_ < 1, 0 < *γ* < 1, and *ϕ* > 0 are the distribution parameters, *c* = 1 − *α*_0_(1 − *γ*) − *α*_1_ *γ*, *μ* = *γ*(1 − *α*_1_)/*c*, and B(y;μ,ϕ) is the beta density function given by [11]

$$\begin{array}{c} B\left(y;\mu ,\phi \right)=\frac{\Gamma (\phi )}{\Gamma (\mu \phi )\Gamma ((1-\mu )\phi )}y^{\mu \phi -1}{\left(1-y\right)}^{(1-\mu )\phi -1},\;0<y<1, \end{array}$$

where 0 < *μ* < 1, *ϕ* > 0, and Γ(⋅) is the gamma function. Here, *ϕ* is a precision parameter (inversion of the dispersion), E(*y*) = *γ*, and $\mathrm{Var}\left(y\right)=\frac{\left(1+\alpha_{1}\phi \right)}{1+\phi }\gamma +(\frac{{\left(1-\alpha_{1}\right)}^{2}\phi }{(1-\alpha_{0}\left(1-\gamma \right)-\alpha_{1}\gamma )\left(1+\phi \right)}-1)\gamma^{2}$. Density (1) is said to be zero and one inflated beta, i.e., $y∼BIm(\alpha_{0},\alpha_{1},\gamma ,\phi )$. Note that *P*(*y* = 0) = *α*_0_(1 − *γ*) and *P*(*y* = 1) = *α*_1_ *γ*, thus if *α*_0_ = 0 and *α*_1_ > 0, the distribution in (1) is called one inflated beta distribution. Differently, if *α*_0_ > 0 and *α*_1_ = 0, the distribution given in (1) is called zero inflated beta distribution.

Let *y*_1_, …, *y*_*n*_ be independent random variables where each *y*_*t*_ has the density in (1) for *t* = 1, …, *n*. The inflated beta regression model with varying dispersion, which is an extension of the regression model proposed by [12], is given by the following structures for modeling the response *y*_*t*_

$$\begin{array}{cc} g_{1}\left(\alpha_{0t}\right) & ={\tilde{x}}_{t}^{⊤}\omega , \end{array}$$ (2)

$$\begin{array}{cc} g_{2}\left(\alpha_{1t}\right) & ={\overset{ˇ}{x}}_{t}^{⊤}\kappa , \end{array}$$ (3)

$$\begin{array}{cc} g_{3}\left(\gamma_{t}\right) & =x_{t}^{⊤}\beta , \end{array}$$ (4)

$$\begin{array}{cc} g_{4}\left(\phi_{t}\right) & ={\ddot{x}}_{t}^{⊤}\zeta , \end{array}$$ (5)

where $\omega ={\left[\omega_{0},\omega_{1},\ldots ,\omega_{k_{1}}\right]}^{⊤}$, $\kappa ={\left[\kappa_{0},\kappa_{1},\ldots ,\kappa_{k_{2}}\right]}^{⊤}$, $\beta ={\left[\beta_{0},\beta_{1},\ldots ,\beta_{k_{3}}\right]}^{⊤}$, and $\zeta ={\left[\zeta_{0},\zeta_{1},\ldots ,\zeta_{k_{4}}\right]}^{⊤}$ are vectors of unknown regression parameters, ${\tilde{x}}_{t}={\left[1,{\tilde{x}}_{t1},\ldots ,{\tilde{x}}_{tk_{1}}\right]}^{⊤}$, ${\overset{ˇ}{x}}_{t}={\left[1,{\overset{ˇ}{x}}_{t1},\ldots ,{\overset{ˇ}{x}}_{tk_{2}}\right]}^{⊤}$, $x_{t}={\left[1,x_{t1},\ldots ,x_{tk_{3}}\right]}^{⊤}$, and ${\ddot{x}}_{t}={\left[1,{\ddot{x}}_{t1},\ldots ,{\ddot{x}}_{tk_{4}}\right]}^{⊤}$ are observations on *k*_1_, *k*_2_, *k*_3_, and *k*_4_ covariates, respectively, and $g_{1}:\left(0,1\right)\to I\;R$, $g_{2}:\left(0,1\right)\to I\;R$, $g_{3}:\left(0,1\right)\to I\;R$, and $g_{4}:\left(0,\infty \right)\to I\;R$ are real-valued link functions with continuous second derivatives. For *g*_1_(⋅), *g*_2_(⋅), and *g*_3_(⋅), several different link functions can be used, such as logit, probit, log-log, complementary log-log, or Cauchy. For *g*_4_(⋅), the choices are log or square root link functions, for example. More details about link functions on the class of beta regression models can be found in [11] and [21].

Notably, the model proposed by [12] assume that, for *t* = 1, …, *n*, *ϕ*_*t*_ = *ϕ* (constant precision). In the present study, the precision parameter is allowed to vary across observations, making the proposed model more general than the original inflated beta regression model. The assumption of non-constant dispersion is natural in several production processes [22–24]. In practical situations, it is important to monitor the dispersion of the process because an increase in dispersion may indicate process deterioration, while a reduction in dispersion means an improvement in process capability [25]. In addition, it is possible to consider control variables for modeling the parameters *α*_0_ and *α*_1_, which are related to the probabilities of zero and one, respectively.

### 2.2 Model-based control chart limits

The purpose of IBRCC is to monitor double bounded processes that contain values equal to zero or one, considering that the mean, precision, and parameters related to the probabilities of zero and one (*α*_0_ and *α*_1_) of the quality characteristic of interest are affected by control variables. Let (1 − *α*) be a control region where *α* is the type I error probability, the lower control limit (LCL), center line (CL), and upper control limit (UCL) of the proposed control chart are defined, respectively, by

$$\begin{array}{cc} {\text{LCL}}_{t} & =F^{-1}(\alpha /2;\alpha_{0t},\alpha_{1t},\gamma_{t},\phi_{t}),\\ {\text{CL}}_{t} & =\gamma_{t},\\ {\text{UCL}}_{t} & =F^{-1}(1-\alpha /2;\alpha_{0t},\alpha_{1t},\gamma_{t},\phi_{t}), \end{array}$$

where $F\left(y\right)=P\left(Y\leq y\right)=\int_{0}^{y}f\left(u,\alpha_{0},\alpha_{1},\gamma ,\phi \right)du$ is the inflated beta cumulative distribution function and *F*^−1^(⋅) is the quantile function of the inflated beta variable. The parameters *α*_0*t*_, *α*_1*t*_, *γ*_*t*_, and *ϕ*_*t*_ are functions of *ω*, *κ*, *β*, and *ζ*, respectively, and through (2), (3), (4), and (5) we have $\alpha_{0t}=g_{1}^{-1}\left({\tilde{x}}_{t}^{⊤}\omega \right)$, $\alpha_{1t}=g_{2}^{-1}\left({\overset{ˇ}{x}}_{t}^{⊤}\kappa \right)$, $\gamma_{t}=g_{3}^{-1}\left(x_{t}^{⊤}\beta \right)$, and $\phi_{t}=g_{4}^{-1}\left({\ddot{x}}_{t}^{⊤}\zeta \right)$. In practice, the model parameters are unknown and estimation methods are necessary to estimate the in-control limits. Thus, we consider the likelihood theory [26–28], which we discuss in the following subsection. We presented results of the log-likelihood and score functions, which are extensions of those developed for the inflated beta regression model proposed by [12].

#### 2.2.1 Likelihood inference

We shall consider the maximum likelihood estimator (MLE) for the parameter vector ***θ*** = (***ω***^⊤^, ***κ***^⊤^, ***β***^⊤^, ***ζ***^⊤^)^⊤^. The log-likelihood function is given by

$$\begin{array}{c} \ell \left(\theta \right)=\sum_{t=1}^{n}\ell_{t}\left(\alpha_{0t},\alpha_{1t},\gamma_{t},\phi_{t}\right), \end{array}$$ (6)

with

$$\begin{array}{cc} \ell (\theta ) & =\sum_{t=1}^{n}\log\left[\alpha_{0t}\left(1-\gamma_{t}\right)\right]1_{0}\left(y_{t}\right)+\log\left(\alpha_{1t}\gamma_{t}\right)1_{1}\left(y_{t}\right)\\ & +\left[\log\left(c_{t}\right)+\log\left(B\left(y_{t};\mu_{t},\phi_{t}\right)\right)\right]\left[\left(1-1_{0}\left(y_{t}\right)\right)\left(1-1_{1}\left(y_{t}\right)\right)\right], \end{array}$$

where $1_{0}\left(y_{t}\right)$ is an indicator function that equals 1 if *y* = 0 and 0 if *y* ∈ (0, 1], $1_{1}\left(y_{t}\right)$ is an indicator function that equals 1 if *y* = 1 and 0 if *y* ∈ [0, 1), and

$$\begin{array}{cc} \log[B\left(y_{t};\mu_{t},\phi_{t}\right)] & =\log\Gamma \left(\phi_{t}\right)-\log\Gamma \left(\mu_{t}\phi_{t}\right)-\log\Gamma \left(\left(1-\mu_{t}\right)\phi_{t}\right)+\left(\mu_{t}\phi_{t}-1\right)y_{t}^{*}+\left(\phi_{t}-2\right)y_{t}^{†}, \end{array}$$

in which *α*_0*t*_, *α*_1*t*_, *γ*_*t*_, and *ϕ*_*t*_ are given by the regression structures in (2), (3), (4), and (5), respectively. Additionally,

$$\begin{array}{cc} y_{t}^{*} & =\{\begin{array}{cc} \log(\frac{y_{t}}{1-y_{t}}), & y_{t}\in \left(0,1\right),\\ 0, & y_{t}=0\;\text{or}\;y_{t}=1, \end{array}\\ \text{and}\;y_{t}^{†} & =\{\begin{array}{cc} \log(1-y_{t}), & y_{t}\in \left(0,1\right),\\ 0, & y_{t}=0\;\text{or}\;y_{t}=1. \end{array} \end{array}$$

By deriving the log-likelihood function in (6) with respect to each element of the parameter vector ***θ***, we obtain the score vector given by *U*(***θ***) = (*U*_*ω*_(***θ***)^⊤^, *U*_*κ*_(***θ***)^⊤^, *U*_*β*_(***θ***)^⊤^, *U*_*ζ*_(*γ*)^⊤^)^⊤^. The MLE of ***θ*** is obtained by solving the non-linear system (*U*_*ω*_(***θ***)^⊤^, *U*_*κ*_(***θ***)^⊤^, *U*_*β*_(***θ***)^⊤^, *U*_*ζ*_(*γ*)^⊤^) = **0**, where **0** is a null vector of dimension (*k*_1_ + *k*_2_ + *k*_3_ + *k*_4_). The MLEs cannot be expressed in closed-form, hence the maximization of the log-likelihood function needs to be numerically conducted through a Newton or quasi-Newton algorithm. In this work, we used the quasi-Newton Broyden-Fletcher-Goldfarb-Shanno (BFGS) method [29] for computational implementation.

The Fisher information matrix (*K*(***θ***)), which is useful for large sample inferences, requires the expectations of second order derivatives of the log-likelihood function. The score vector *U*(***θ***) and Fisher’s information matrix can be found in the Appendix.

To test hypotheses on the parameter ***θ***_*j*_, *j* = 1, …, (*k*_1_ + *k*_2_ + *k*_3_ + *k*_4_), we consider the null hypothesis $H_{0}:\theta_{j}=\theta_{j}^{0}$ versus $H_{1}:\theta_{j}\neq \theta_{j}^{0}$. The Wald test may be considered using the following *z* statistic [26]

$$\begin{array}{c} z=\frac{{\hat{\theta }}_{j}-\theta_{j}^{0}}{\hat{\text{se}}\left({\hat{\theta }}_{j}\right)}, \end{array}$$

where ${\hat{\theta }}_{j}$ represents the MLE of ***θ***_*j*_ and the standard error of ${\hat{\theta }}_{j}$ is given by $\hat{\text{se}}\left({\hat{\theta }}_{j}\right)={\left[\text{diag}\left(\hat{\text{cov}}\left(\hat{\theta }\right)\right)\right]}_{j}^{1/2}$, in which $\hat{\text{cov}}\left(\hat{\theta }\right)=K^{-1}\left(\hat{\theta }\right)$ is the asymptotic variance and covariance matrix of $\hat{\theta }$. In large sample sizes and under $H_{0}$, the *z* statistic follows a standard normal distribution [26]. The test is performed by comparing the computed *z* statistic with the usual quantiles of the standard normal distribution.

#### 2.2.2 Control limits estimation

After obtaining the MLE of ***θ***, $\hat{\theta }={\left({\hat{\omega }}^{⊤},{\hat{\kappa }}^{⊤},{\hat{\beta }}^{⊤},{\hat{\zeta }}^{⊤}\right)}^{⊤}$, and considering an in-control process, the estimated control limits are given by

$$\begin{array}{cc} {\hat{\text{LCL}}}_{t} & =F^{-1}(\alpha /2;{\hat{\alpha }}_{0t},{\hat{\alpha }}_{1t},{\hat{\gamma }}_{t},{\hat{\phi }}_{t}), \end{array}$$ (7)

$$\begin{array}{cc} {\hat{\text{CL}}}_{t} & ={\hat{\gamma }}_{t}, \end{array}$$

$$\begin{array}{cc} {\hat{\text{UCL}}}_{t} & =F^{-1}(1-\alpha /2;{\hat{\alpha }}_{0t},{\hat{\alpha }}_{1t},{\hat{\gamma }}_{t},{\hat{\phi }}_{t}), \end{array}$$ (8)

where ${\hat{\alpha }}_{0t}=g_{1}^{-1}\left({\tilde{x}}_{t}^{⊤}\hat{\omega }\right)$, ${\hat{\alpha }}_{1t}=g_{2}^{-1}\left({\overset{ˇ}{x}}_{t}^{⊤}\hat{\kappa }\right)$, ${\hat{\gamma }}_{t}=g_{3}^{-1}\left(x_{t}^{⊤}\hat{\beta }\right)$, and ${\hat{\phi }}_{t}=g_{4}^{-1}\left({\ddot{x}}_{t}^{⊤}\hat{\zeta }\right)$. Thus, we propose the following algorithm to implement the IBRCC.

1. Fit the inflated beta regression model with varying dispersion under Phase I and obtain the MLEs, namely $\hat{\omega }$, $\hat{\kappa }$, $\hat{\beta }$, and $\hat{\zeta }$.
2. Using covariates in Phase II, estimate *α*_0*t*_, *α*_1*t*_, *γ*_*t*_, and *ϕ*_*t*_ such that ${\hat{\alpha }}_{0t}=g_{1}^{-1}\left({\tilde{x}}_{t}^{⊤}\hat{\omega }\right)$, ${\hat{\alpha }}_{1t}=g_{2}^{-1}\left({\overset{ˇ}{x}}_{t}^{⊤}\hat{\kappa }\right)$, ${\hat{\gamma }}_{t}=g_{3}^{-1}\left(x_{t}^{⊤}\hat{\beta }\right)$, and ${\hat{\phi }}_{t}=g_{4}^{-1}\left({\ddot{x}}_{t}^{⊤}\hat{\zeta }\right)$.
3. For a given type I error probability *α* and the estimates ${\hat{\alpha }}_{0t}$, ${\hat{\alpha }}_{1t}$, ${\hat{\gamma }}_{t}$, and ${\hat{\phi }}_{t}$, compute the estimated control limits using (7) and (8).
4. Plot each data point *y*_*t*_ together with the estimated control limits ${\hat{\text{UCL}}}_{t}$ and ${\hat{\text{LCL}}}_{t}$, for *t* = 1, …, *n*.

The observation *y*_*t*_ that is out of the estimated control limits interval (${\hat{\text{UCL}}}_{t},{\hat{\text{LCL}}}_{t})$ is considered out-of-control.

## 3 Simulation study

This section presents a Monte Carlo simulation study to evaluate the estimators of the introduced inflated beta regression model with varying dispersion and the performance of the IBRCC. The performance of the proposed control chart is compared with some alternatives in literature, namely: the usual linear regression control chart (RCC) [1], the beta regression control chart (BRCC) [6], and the inflated beta control chart (IBCC) [30]. Note that the RCC is a classical regression control chart that works under Gaussian assumptions. The other control charts are state-of-the-art alternatives, but the BRCC does not consider inflation in zeros and/or ones and the IBCC does not include covariates.

We used the following structures for data generation

$$\begin{array}{cc} \text{logit}(\alpha_{0t}) & =\omega_{0}+\omega_{1}{\tilde{x}}_{t},\\ \text{logit}(\alpha_{1t}) & =\eta_{0}+\eta_{1}{\overset{ˇ}{x}}_{t},\\ \text{logit}(\gamma_{t}) & =\beta_{0}+\beta_{1}x_{t},\\ \text{log}(\phi_{t}) & =\zeta_{0}+\zeta_{1}{\ddot{x}}_{t}, \end{array}$$

with *t* = 1, …, *n*. The values of ${\tilde{x}}_{t}$, ${\overset{ˇ}{x}}_{t}$, and ${\ddot{x}}_{t}$ were obtained from a Bernoulli distribution with parameter *p* = 0.3, and *x*_*t*_ was generated from a uniform distribution in the interval (0, 1), thus considering discrete and continuous random variables. We considered 5, 000 Monte Carlo replications and sample sizes *n* = 100, 200, and 500. According to [31] and [32], this number of replications is enough to obtain accurate results. All simulations were performed using the R programming language [33].

In the numerical evaluation, we considered several scenarios with different characteristics, namely: zero and one inflated beta regression model (Scenario 1), zero inflated beta regression model (Scenarios 2, 4, and 6), and one inflated beta regression model (Scenarios 3, 5, and 7). The parameter values are shown in Table 1. In Scenario 1, the mean is centered on the standard unit interval, *γ* ∈ [0.43, 0.57], and the average percentage of zeros and ones in the sample is approximately equal to 13% for both. Scenarios 2, 4, and 6 consider the mean close to zero, with *γ* ∈ [0.063, 0.154], [0.012, 0.039], and [0.039, 0.119], and average percentages of zeros in the sample equal to 9.3%, 3.4%, and 6.4%, respectively. For Scenarios 3, 5, and 7, the mean is close to one, with *γ* ∈ [0.881, 0.971], [0.668, 0.924], and [0.858, 0.952], and average percentages of ones in the sample equal to 8.3%, 2.6%, and 23.5%, respectively.

**Table 1:** Different scenarios considered in the simulation study.

Scenario	Parameters							
	*ω*_0_	*ω*_1_	*η*_0_	*η*_1_	*β*_0_	*β*_1_	*ζ*_0_	*ζ*_1_
1	−1.00	−0.20	−1.00	−0.20	−0.30	0.60	2.00	1.00
2	−2.30	0.90	0.00	0.00	−2.40	0.80	4.50	−0.30
3	0.00	0.00	−2.50	0.50	3.50	−1.50	2.00	−0.70
4	−3.50	0.50	0.00	0.00	−4.40	1.20	5.50	−0.50
5	0.00	0.00	−3.50	0.30	2.50	−1.80	1.00	−0.20
6	−2.50	0.90	0.00	0.00	−2.70	1.00	3.00	−0.30
7	0.00	0.00	−1.00	−0.20	3.00	−1.20	1.50	−0.30

### 3.1 Point estimation evaluation

For the point estimation evaluation, we computed the mean, percentage relative bias (RB), and mean square error (MSE) for each estimator in all Scenarios (see Table 1). For brevity and similarity of results, we only present results for Scenarios 1, 2, and 7 (*n* = 100 and *n* = 500) as shown in Table 2. The figures show that the mean of the estimators is close to the corresponding parameter values. The RB and MSE decrease when the sample size increases, indicating that the MLEs are consistent. For instance, for *β*_1_ (*γ* submodel) in Scenario 1, the RB of the estimator is equal to 0.2257% for *n* = 100 and equal to −0.0548% for *n* = 500. Regarding MSE, considering *ω*_0_ in Scenario 2 and *n* ∈ {100, 500}, the MSE is equal to 0.2291 and 0.0373, respectively. As in other studies related to beta regression [21, 34], it is noteworthy that the RB of MLEs corresponding to the precision covariate parameters is greater than those of that model the mean response. For instance, consider ${\hat{\zeta }}_{1}$ (*ϕ* submodel) in Scenario 7, we have RB = −8.2035% for *n* = 100 and RB = −2.4411% for *n* = 500. Regarding parameters related to the probabilities of zeros and ones, the bias also decreases considerably as sample size increases. For example, in Scenario 1 and *n* = 100, the estimator of *ω*_1_ (*α*_0_ submodel) yields RB = 16.9130% and the estimator of *η*_1_ (*α*_1_ submodel) yields RB = 14.8922%. For *n* = 500, the bias for the same estimators reduces to 4.5210% and 3.4431%, respectively.

**Table 2:** Monte Carlo simulation results of point estimation evaluation.

Scenario 1									
*n*↓	Param. →	*ω*_0_ = −1.00	*ω*_1_ = −0.20	*η*_0_ = −1.00	*η*_1_ = −0.20	*β*_0_ = −0.30	*β*_1_ = 0.60	*ζ*_0_ = 2.00	*ζ*_1_ = 1.00
100	Mean	−1.0393	−0.2338	−1.0439	−0.2298	−0.3026	0.6013	2.0455	1.0717
	RB	3.9304	16.9130	4.3925	14.8922	0.8683	0.2257	2.2770	7.1753
	MSE	0.1396	0.3936	0.1425	0.4091	0.0279	0.0553	0.0403	0.1412
500	Mean	−1.0078	−0.2090	−1.0146	−0.2069	−0.3009	0.5997	2.0080	1.0142
	RB	0.7846	4.5210	1.4628	3.4431	0.3143	−0.0548	0.4141	1.4201
	MSE	0.0230	0.0695	0.0241	0.0686	0.0052	0.0101	0.0071	0.0250
Scenario 2									
*n*↓	Param. →	*ω*_0_ = −2.30	*ω*_1_ = 0.90	*η*_0_ = 0.0	*η*_1_ = 0.0	*β*_0_ = −2.40	*β*_1_ = 0.80	*ζ*_0_ = 4.50	*ζ*_1_ = −0.30
100	Mean	−2.3694	0.9302	—	—	−2.4034	0.80115	4.5469	−0.2616
	RB	3.0207	3.3572	—	—	0.1455	0.1445	1.0435	−12.7889
	MSE	0.2291	0.2588	—	—	0.0076	0.0161	0.0359	0.1254
500	Mean	−2.313	0.9030	—	—	2.4002	0.8005	4.5093	−0.2911
	RB	0.5780	0.3328	—	—	0.0096	0.0637	0.2072	−2.9518
	MSE	0.0373	0.0403	—	—	0.0013	0.0027	0.0062	0.0222
Scenario 7									
*n*↓	Param.→	*ω*_0_ = 0.0	*ω*_1_ = 0.0	*η*_0_ = −1.00	*η*_1_ = −0.20	*β*_0_ = 3.00	*β*_1_ = −1.20	*ζ*_0_ = 1.50	*ζ*_1_ = −0.30
100	Mean	—	—	−1.0201	-0.2602	3.0223	-1.2099	1.5475	−0.2753
	RB	—	—	2.0136	30.1092	0.7455	0.8254	3.1698	−8.2035
	MSE	—	—	0.0827	0.3659	0.0952	0.2023	0.0478	0.0990
500	Mean	—	—	−1.0007	−0.2116	3.0062	−1.2028	1.5094	−0.2927
	RB	—	—	0.0794	5.8189	0.2071	0.2380	0.6269	−2.4411
	RB	—	—	0.0156	0.0539	0.0155	0.0318	0.0085	0.0176

In practice, the regression model relating the output and covariates is rarely known and the parameters have to be estimated. Our simulation results show that the MLE in the proposed model perform well, presenting low MSE for the estimates in all situations. This way, the proposed control chart may also present good performance in practice. In the next section, we shall investigate the run length performance of the IBRCC with estimated parameters.

### 3.2 Control charts performance

This section presents a run length analysis to evaluate the performance of the considered control charts. When the process is in-control, the run length (RL) distribution follows a geometric distribution with parameter *α*, which is the type I error probability [35]. The ability of a control chart to detect changes in the process is usually measured by the average number of observations until the detection of an out-of-control point (ARL) [36]. However, other measures can also be used for this purpose. We considered another location measure, the median (MRL), a dispersion measure, and the standard deviation (SDRL) of the RL distribution. Additionally, we computed the mean absolute percentage error (MAPE) for each measure for all evaluated control charts.

We compared the proposed IBRCC with the standard RCC [1], and the state-of-the-art charts, namely BRCC [6] and IBCC [30]. Since the BRCC does not consider values equal to zero or one, we replaced zeros by 0.0001 and ones by 0.9999 for its application. For all considered control charts, we examined two aspects of evaluation: in-control (${\text{ARL}}_{0}=\frac{1}{\alpha }$, ${\text{MRL}}_{0}=\frac{\ln(0.5)}{\ln(1-\alpha )}$, ${\text{SDRL}}_{0}=\sqrt{\frac{\left(1-\alpha \right)}{\alpha^{2}}}$) and out-of-control (${\text{ARL}}_{1}=\frac{1}{1-\beta }$, where *β* is the type II error probability) [30, 35]. For brevity, we do not present the MRL and SDRL results for the out-of-control process. The control charts were evaluated in Scenarios 2 to 7 (Table 2), considering inflation in 0 or 1. Scenario 1 was not covered in this section because it does not reflect real statistical process control situations, being possible to present perfect nonconforming and perfect conforming in the same process.

To ensure that the comparisons between ARL_1_ occur between control charts of same ARL_0_, we adjusted the chart limits to obtain ARL_0_ equal to the specified nominal values of 100 and 370. This control chart calibration is suggested in the literature [30, 37–39]. After ARL_0_ calibration, a *δ* change was induced in the mean and precision regression structures to generate out-of-control processes as the following: logit(*γ*_*t*_) = *δ* + *β*_0_ + *β*_1_ *x*_*t*_ and $\text{log}\left(\phi_{t}\right)=\delta +\zeta_{0}+\zeta_{1}\ddot{x_{t}}$. By enabling the process to be out-of-control, we obtained the estimated ARL_1_ for different values of *δ*. When *δ* = 0, the process is in-control and the ARL_0_ can be evaluated.

The ${\hat{\text{ARL}}}_{0}$, ${\hat{\text{MRL}}}_{0}$, and ${\hat{\text{SDRL}}}_{0}$ evaluation results are shown in Tables 3 and 4. Consider an in-control process with *α* − values of 0.01 and 0.0027, from the geometric distribution of the RL we have ARL values equal to 100 and 370, nominal MRL values equal to 69.0 and 256.1, and values of SDRL equal to 99.5 and 369.5, respectively. The IBRCC showed better performance than BRCC, RCC, and IBCC, reaching empirical values closer to the nominal levels in all evaluated scenarios. In Scenarios 2, 4, and 6, the IBRCC and IBCC obtained 0 as the lower control limit, thus no point exceeded this limit. Similarly, in Scenarios 3, 5, and 7, the upper control limit of the mentioned charts were 1. The fact that these scenarios present the 0 or 1 as control limits is related to the value of the probabilities of occurrence of 0 or 1. That is, the IBRCC and IBCC will present zero as control limit when $P\left(Y=0\right)={\hat{\alpha }}_{0t}\left(1-{\hat{\gamma }}_{t}\right)\geq \alpha /2$ and one as control limit when $P\left(Y=1\right)={\hat{\alpha }}_{1t}{\hat{\gamma }}_{t}\geq \alpha /2$. The high probability of *Y* assuming values equal to one or zero means that these values are not atypical (out of control) but usual occurrences of the process.

**Table 3:** Run length analysis to evaluate the IBRCC, BRCC, RCC, and IBCC with *α* = 0.01.

Scenario	*n*	Control Chart											
		IBRCC			BRCC			RCC			IBCC		
		${\hat{\text{MRL}}}_{0}$	${\hat{\text{ARL}}}_{0}$	${\hat{\text{SDRL}}}_{0}$	${\hat{\text{MRL}}}_{0}$	${\hat{\text{ARL}}}_{0}$	${\hat{\text{SDRL}}}_{0}$	${\hat{\text{MRL}}}_{0}$	${\hat{\text{ARL}}}_{0}$	${\hat{\text{SDRL}}}_{0}$	${\hat{\text{MRL}}}_{0}$	${\hat{\text{ARL}}}_{0}$	${\hat{\text{SDRL}}}_{0}$
2	100	69.48	99.43	97.75	7.54	10.65	10.12	43.53	62.19	61.17	77.08	109.87	107.91
	200	69.16	98.73	97.46	7.58	10.69	10.17	38.34	54.85	54.04	76.69	109.59	108.19
	500	69.42	99.28	97.67	7.57	10.69	10.16	37.96	54.16	53.32	77.53	110.57	108.69
3	100	74.27	106.22	104.76	7.68	10.86	10.34	23.62	33.78	33.09	87.16	124.41	122.26
	200	70.87	101.17	99.52	7.79	11.01	10.50	21.62	30.85	30.21	86.61	122.65	119.98
	500	70.24	100.49	98.76	7.87	11.10	10.57	21.42	30.58	29.93	88.00	125.35	122.89
4	100	63.05	90.08	88.78	19.24	27.48	26.92	35.35	50.50	49.66	97.63	138.71	135.74
	200	68.37	97.81	96.37	19.95	28.52	27.96	30.22	43.21	42.53	95.68	136.66	134.42
	500	69.22	98.90	97.20	20.33	29.04	28.42	29.84	42.51	41.75	97.63	139.37	137.04
5	100	66.68	94.64	92.66	19.00	27.16	26.56	32.33	46.20	45.30	93.19	132.47	129.93
	200	69.76	99.76	98.21	19.73	28.20	27.61	27.38	39.18	38.55	92.42	132.07	129.97
	500	69.79	100.31	100.08	20.24	28.91	28.30	26.02	37.16	36.47	92.98	132.53	129.88
6	100	69.48	99.03	97.10	10.04	14.24	13.71	27.49	39.24	38.47	79.40	113.00	110.72
	200	70.71	101.42	100.22	10.20	14.48	13.94	25.08	35.84	35.20	78.84	112.44	110.78
	500	70.12	100.28	98.59	10.29	14.61	14.06	24.84	35.49	34.80	80.02	114.04	112.06
7	100	75.14	105.57	103.76	3.00	4.17	3.64	21.89	31.30	30.71	82.76	118.03	116.25
	200	71.22	101.62	100.05	3.00	4.18	3.64	20.32	29.03	28.45	82.43	117.38	115.39
	500	69.88	99.88	98.28	3.00	4.19	3.65	19.97	28.52	27.90	82.88	118.20	115.92
MAPE	100	4.73	4.77	5.23	84.05	84.24	84.71	55.82	56.13	56.72	24.03	22.75	21.07
	200	1.45	1.32	1.30	83.63	83.82	84.28	60.92	61.17	61.64	22.94	21.80	20.39
	500	0.57	0.50	1.27	83.38	83.58	84.06	61.62	61.93	62.45	24.47	23.34	21.69

**Table 4:** Run length analysis to evaluate the IBRCC, BRCC, RCC, and IBCC with *α* = 0.0027.

Scenario	*n*	Control Chart											
		IBRCC			BRCC			RCC			IBCC		
		${\hat{\text{MRL}}}_{0}$	${\hat{\text{ARL}}}_{0}$	${\hat{\text{SDRL}}}_{0}$	${\hat{\text{MRL}}}_{0}$	${\hat{\text{ARL}}}_{0}$	${\hat{\text{SDRL}}}_{0}$	${\hat{\text{MRL}}}_{0}$	${\hat{\text{ARL}}}_{0}$	${\hat{\text{SDRL}}}_{0}$	${\hat{\text{MRL}}}_{0}$	${\hat{\text{ARL}}}_{0}$	${\hat{\text{SDRL}}}_{0}$
2	100	279.42	377.20	346.43	7.59	10.72	10.19	87.81	124.47	121.69	300.24	416.27	396.89
	200	266.36	368.23	350.79	7.61	10.73	10.20	73.39	105.73	104.39	293.99	411.46	396.08
	500	262.62	366.62	349.45	7.59	10.71	10.18	72.33	103.34	101.68	301.16	419.35	402.25
3	100	318.25	426.92	387.41	8.17	11.59	11.07	35.13	50.12	49.23	305.31	422.34	401.87
	200	279.92	385.53	364.98	8.23	11.68	11.16	31.33	44.70	43.90	299.12	415.33	395.29
	500	270.43	376.94	361.74	8.26	11.70	11.17	30.91	44.12	43.37	309.09	428.96	410.88
4	100	250.95	342.62	319.09	20.10	28.74	28.17	88.01	124.20	120.69	444.32	607.52	570.76
	200	261.78	364.01	348.01	20.42	29.19	28.62	71.42	101.93	100.16	434.44	596.99	564.95
	500	261.74	364.58	348.34	20.51	29.31	28.70	69.61	99.46	97.80	448.49	616.69	581.46
5	100	258.92	351.89	327.00	23.40	33.48	32.84	115.84	157.55	146.89	405.71	555.36	524.39
	200	278.17	382.32	358.68	24.04	34.35	33.72	73.29	103.69	100.90	399.52	551.36	523.64
	500	279.66	383.90	356.75	24.40	34.89	34.24	62.04	88.66	87.15	404.63	557.51	528.28
6	100	279.43	379.74	353.87	10.69	15.18	14.64	43.98	62.72	61.53	274.89	383.03	366.43
	200	280.08	386.16	365.02	10.78	15.29	14.75	39.10	55.86	55.04	270.35	378.39	364.49
	500	267.76	373.17	356.16	10.79	15.33	14.78	38.57	54.98	54.06	276.81	387.16	372.06
7	100	312.39	417.06	376.00	3.00	4.23	3.70	35.12	50.18	49.31	335.56	466.47	445.26
	200	286.76	390.91	365.49	3.00	4.24	3.70	31.35	44.82	44.10	333.98	463.49	443.27
	500	268.50	373.47	358.37	3.00	4.24	3.70	30.46	43.55	42.81	341.14	471.54	448.48
MAPE	100	11.26	7.50	7.04	95.25	95.32	95.46	73.59	74.36	75.22	34.45	28.42	22.32
	200	7.58	3.27	2.89	95.18	95.25	95.39	79.18	79.43	79.77	32.20	26.89	21.68
	500	4.82	1.63	3.89	95.15	95.22	95.36	80.22	80.45	80.75	35.45	29.78	23.74

Tables 3 and 4 also show the MAPE results. The proposed control chart has the lowest values for the MAPE. For example, consider *α* = 0.01, corresponding ARL_0_, and *n* = 200, the MAPE obtained for the IBRCC, BRCC, RCC, and IBCC were, 1.32, 83.82, 61.17, and 21.80, respectively. It is noteworthy that, for the IBRCC, the MAPE decreases considerably when the sample size increases.

Among the considered alternative control charts, the IBCC achieved better performance than the BRCC and RCC in all scenarios. In Scenario 2 (Table 4), for *n* = 200, the IBCC presented a false alarm after 411 samples, when, in fact, a false alarm was expected for every 370 samples. In the same scenario, the BRCC and the RCC presented a false alarm rate in approximately 11 and 106 samples, respectively. These results show the importance of considering an accurate model to reduce false alarms. We also note that the BRCC obtained the worst performance. In Table 3, consider Scenario 3 and *n* = 100, the RCC and BRCC presented a false alarm in approximately each 50 and 27 observations, respectively. It is important to note that BRCC performance worsened as the 0 or 1 percentage increased. Confirming this fact, the IBCC also presented lower MAPE than BRCC and RCC in all scenarios. Considering *α* = 0.0027, *n* = 500, and the MRL_0_ measure, the MAPE obtained for the IBRCC, BRCC, RCC, and IBCC were, respectively, 4.82, 95.15, 80.22, and 35.45.

**Fig 1:**
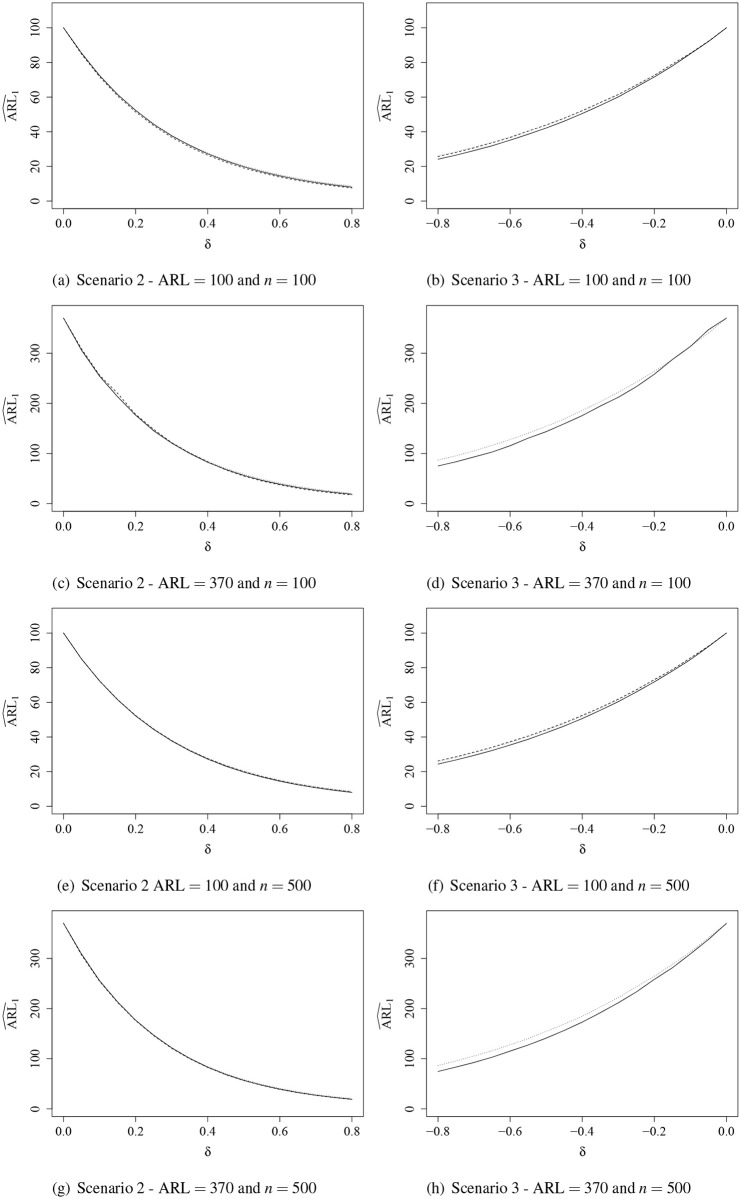
${\hat{\text{ARL}}}_{1}$ curves evaluation for the inflated beta regression control chart (solid line), regression control chart (dashed line), and inflated beta control chart (dotted line) when the mean is out-of-control.

**Fig 2:**
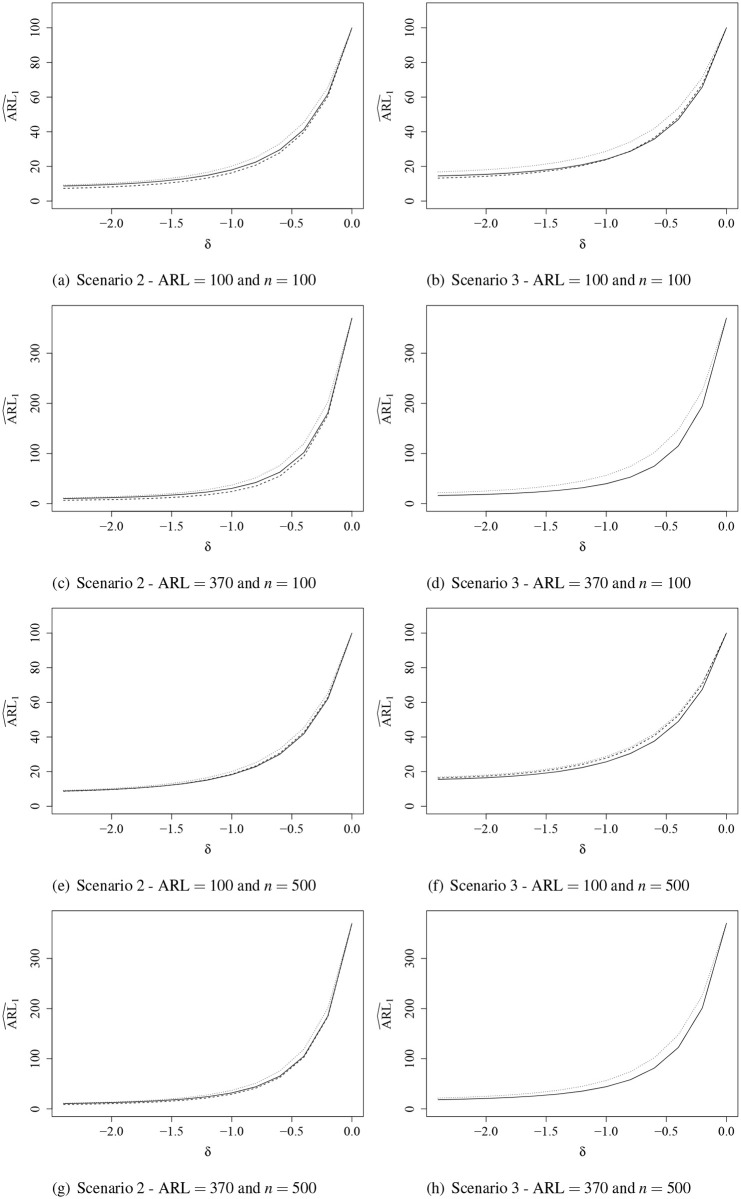
${\hat{\text{ARL}}}_{1}$ curves evaluation for the inflated beta regression control chart (solid line), regression control chart (dashed line), and inflated beta control chart (dotted line) when the precision is out-of-control.

Results of the ARL_1_ evaluation are shown graphically in Figs 1 and 2. It was not possible to correct ARL_0_ for the BRCC due to the poor in-control performance. Thus, the evaluation of ARL_1_ was given only for the IBRCC, RCC, and IBCC. It is noteworthy that when several control charts are compared in terms of ARL, the one that presents the lowest ARL_1_ among those with same ARL_0_ is the control chart that outperforms the competitors [30]. By analyzing the ARL_1_ results, when the perturbation was introduced in the mean of the process (Fig 1), we observe that in Scenario 3 the IBRCC performs better than the RCC and IBCC, and in Scenario 2 the performance of the control charts are similar. We note that the IBRCC detects more quickly the out-of-control process. For example, in Scenario 3, ARL = 370, *n* = 100, and *δ* = −0.4, the IBRCC takes 176 samples on average to detect a change in the process, while the IBCC takes 186 and the RCC takes 192 to detect a change of same magnitude. The simulation results showed similar behavior when a perturbation in the precision of the process occurs (Fig 2). The control charts detect process changes more quickly as the precision increases (dispersion decreases).

By considering the results obtained in the simulation, we see a necessity of using a control chart based on an appropriate regression model, such as the IBRCC, when the variable of interest is restricted to the intervals [0, 1) or (0, 1]. The use of the linear regression-based control chart is inappropriate for data of this type since the support of the usual regression model is the whole real space. Interestingly, the BRCC proved to be more inadequate in the presence of values equal to zero or one than the traditional RCC or the IBCC that uses inflated beta distribution but does not consider a regression structure. Since the BRCC does not accommodate values equal to zero or one, by substituting zeros for 0.0001 and ones for 0.9999, an inflation in these values is induced. That is, the probability mass at 0.0001 and/or 0.9999 exceeds what is allowed by the beta distribution, which is an absolutely continuous distribution. This reflects on the estimates of the parameters of the regression structures and, automatically, the estimates of the control limits are impaired.

## 4 Real data application

This section contains an empirical application in which the proposed control chart (IBRCC) and three other competing control charts are analyzed: the RCC, BRCC, and IBCC. The data evaluated in this section refer to the public administrative efficiency of the municipalities in the state of São Paulo, Brazil. The data are a subset of those analyzed by [40], who considered all Brazilian municipalities. The dataset we used contains 427 municipalities for the year 2000 and it is available at http://www.de.ufpb.br/~luiz/datasets/Dataset_plosone.txt. The covariates are from Secretaria do Tesouro Nacional (http://www.tesouro.fazenda.gov.br/), Instituto Brasileiro de Geografia e Estatística (IBGE) (https://www.ibge.gov.br/), and Instituto de Pesquisa Econômica Aplicada (IPEA) (https://www.ipea.gov.br/portal/), Brazil. The quality characteristic, *y*, is introduced by [40] and represents individual observations of an efficiency index, assuming values in (0, 1] and measuring how well mayors spend taxpayer money in order to provide them with public services. The efficiency index is equal to one when there is full efficiency. There are 32 units that are fully efficient (i.e., about 7.5% of the observations are equal to one). A brief description of the variables used in the analysis is presented in Table 5. Variables *CONS*, *R*2, and *MT* are dummies, i.e., they are equal to 0 or 1. The covariate *CONS* equals 1 if the municipality participates in the inter-municipal consortia, the covariate *R*2 equals 1 whenever the municipality receives more than 10% of its tax revenue to *royalty*, and the covariate *MT* equals 1 whenever the municipality is tourist, 0 otherwise for the three dummies covariates. It is important to mention that 100 municipalities were sorted to estimate the model parameters (Phase I), while the remaining observations were used for monitoring (Phase II).

**Table 5:** Description of the variables for efficiency data.

Variable	Description
*EFFIC*	Efficiency scores
*EXP*	Personnel expenses in Reais
*INC*	Average income in Reais
*CONS*	Participation in inter-municipal consortia
*URB*	Urbanization rate
*R*2	Royalties
*E*20	Demographic density
*MT*	Tourist municipality

At the outset, the inflated (at one) beta mean regression model, the beta regression model substituting 1 for 0.9999, and a linear regression model were selected and fitted. We used the logit link for *γ* and *α*_1_ and the log link for *ϕ*. For the beta regression, we considered logit for *μ* and log link for *ϕ*. The maximum likelihood estimates of the models parameters are displayed in Table 6. All covariates were significant at the nominal level of 5%. In order to compare the fitted regression models, we considered the MAPE and MSE between the observed and fitted values. According to these criteria the inflated beta regression model outperforms the other ones, with MAPE = 26.8835 and MSE = 0.0387, while the beta regression model obtains MAPE = 29.4101 and MSE = 0.0454, and linear regression model achieves MAPE = 29.3617 and MSE = 0.0389.

**Table 6:** Adjusted models for efficiency data.

The fitted inflated beta regression model										
	Submodel for *γ*				Submodel for *ϕ*		Submodel for *α*_0_			
	Intercept	*E*20	*INC*	*URB*	Intercept	*MT*	Intercept	*EXP*	*INC*	*R*2
Estimate	0.6552	0.0001	−0.0015	0.0124	2.0339	0.7744	−2.2551	−1.0427	0.0014	0.5052
Std. error	0.4348	<0.0001	0.0005	0.0059	0.1527	0.3822	0.0884	0.0071	0.0001	0.0987
*p*-value	0.1319	<0.0001	0.0009	0.0371	<0.0001	0.0428	<0.0001	<0.0001	<0.0001	<0.0001
The fitted beta regression model										
	Submodel for *μ*					Submodel for *ϕ*				
	Intercept	*EXP*	*E*20	*URB*	*R*2	Intercept	*CONS*	*URB*		
Estimate	−0.7151	−0.1436	0.0002	0.0171	0.7391	4.2200	−2.2783	−0.0292		
Std. error	0.4272	0.0704	0.0001	0.0056	0.3100	0.8236	0.3102	0.0095		
*p*-value	0.0941	0.0415	0.0280	0.0023	0.0173	<0.0001	<0.0001	0.0022		
The fitted linear regression model										
		Model for *μ*								
	Intercept	*CONS*	*URB*							
Estimate	0.3829	0.1282	0.0031							
Std. error	0.1126	0.0497	0.0013							
*p*-value	0.0010	0.0114	0.0196							

Table 7 presents some descriptive statistics of the estimated control limits. Note that the proposed control chart and the IBCC are the only ones that have an upper control limit constant and equal to one. Differently, when beta regression control chart is used, the control limits were restricted to the open interval (0, 1) and thus, in this case, fully efficient municipalities are considered out-of-control. In addition, we verify that, by using the standard RCC, the limits assume values below zero and above one, not being restricted to the interval (0, 1], where the data are distributed. The interpretation of the limits, in this case, makes no practical sense and leads to loss of detection power of out-of-control points.

**Table 7:** Descriptive statistics—minimum (min), first quantile (*Q*_1/4_), median, mean, third quantile (*Q*_3/4_), and maximum (max)—control limits for efficiency data.

Limits	min	*Q*_1/4_	median	mean	*Q*_3/4_	max
LCL_IBRCC_	0.0042	0.2176	0.2500	0.2546	0.2832	0.5936
UCL_IBRCC_	1.0000	1.0000	1.0000	1.0000	1.0000	1.0000
LCL_BRCC_	0.0001	0.0998	0.1656	0.1429	0.2006	0.4323
UCL_BRCC_	0.0001	0.9582	0.9864	0.9594	0.9974	0.9999
LCL_RCC_	-0.0070	0.1731	0.2064	0.2040	0.2305	0.3640
UCL_RCC_	0.9057	1.0858	1.1191	1.1167	1.1432	1.2767
LCL_IBCC_	0.2424	0.2424	0.2424	0.2424	0.2424	0.2424
UCL_IBCC_	1.0000	1.0000	1.0000	1.0000	1.0000	1.0000

**Fig 3:**
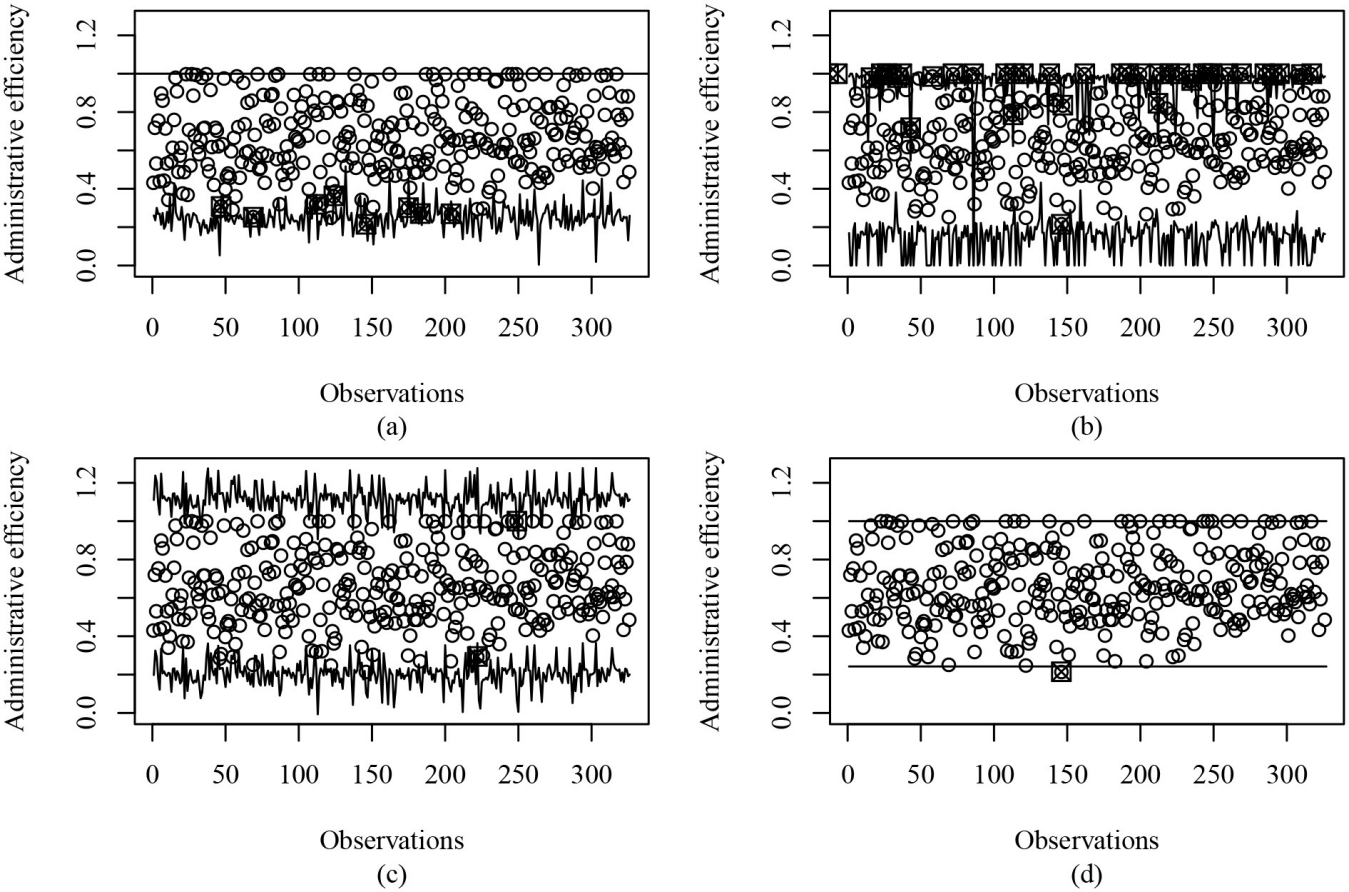
Plot of the control limits based on (a) inflated beta regression control chart, (b) beta regression control chart, (c) regression control chart, and (d) inflated beta control chart for monitoring the efficiency indexes for municipalities in the state of São Paulo, Brazil, considering ARL_0_ = 100.

Fig 3 graphically presents the control limits of the (a) IBRCC, (b) BRCC, (c) RCC, and (d) IBCC together with the observed values of efficiency considering ARL_0_ = 100. Considering the fact that the efficiency index assumes values in (0, 1], the proposed model-based control chart (IBRCC) presents limits with a smaller range. Interestingly, the BRCC does not accommodate values equal to one by substituting values equal to one for 0.9999, an inflation in these values is induced, therefore the BRCC is less adequate in the presence of values equal to one than the traditional RCC. The use of the linear regression-based control chart is inappropriate for data of this type since the support of the usual regression model is the whole real space. Finally, the IBCC that uses inflated beta distribution but does not consider regression structure presents constant limits that are not appropriate in situations were we have control variables (covariates). It is worth mentioning that IBRCC detected 7 out-of-control points, while BRCC detected 36 out-of-control points. Lastly, we carried out the RESET misspecification test [41], where the null hypothesis is that the fitted model is correctly specified and the alternative hypothesis is that there is model misspecification. We perform the test using the second power of the estimated mean linear predictor as testing variables. We do not reject the null hypothesis at the 1% nominal level, thus suggesting that our model is correctly specified.

## 5 Conclusions

In this paper, we proposed a new model-based control chart for controlling quality characteristics limited to the intervals (0, 1] or [0, 1) using the inflated beta regression model. For this purpose, we extended the inflated beta regression model proposed by [12] by allowing a regression structure for the precision parameter. In this way, it is possible to model the mean response, the data precision, and functions of the probability of a given observation assuming zero or one through a regression framework. Our simulation study showed that the relative bias and mean square error decrease when the sample size increases. With regard to the sensitivity analysis in terms of run length (RL), the proposed IBRCC showed the best performance in all considered cases. In addition, the results indicated that it is better to ignore the explanatory variables and use the inflated beta control chart (IBCC) than to use a control chart based on an inappropriate regression model. We also considered an application to real data and highlight the practical importance of the proposed chart when the response is distributed in unit intervals containing ones. Finally, we suggest the use of the inflated beta regression control chart to monitor output quality characteristics, which is better characterized by a functional relation between the response variable, double bounded in unit intervals containing zeros or ones along with one or more explanatory variables.

## A Score function and Fisher’s information matrix

In this appendix we obtain the score function and presented a closed-form expression for Fisher’s information matrix for all parameters of the inflated beta regression model with varying dispersion. We assume that the observed values of the dependent fractional variable are sorted according to the 0, 1, and (0, 1)-values with *n*_0_, *n*_1_, and *n* − *m* terms, respectively, where *m* = *n*_0_ + *n*_1_. Furthermore, $\mu_{t}^{*}=E\left(y_{t}^{*}\right)=\psi \left(\mu_{t}\phi_{t}\right)-\psi \left(\left(1-\mu_{t}\right)\phi_{t}\right)$ and $\mu_{t}^{†}=E\left(y_{t}^{†}\right)=\psi \left(\left(1-\mu_{t}\right)\phi_{t}\right)-\psi \left(\phi_{t}\right)$, where *ψ*(⋅) is the digamma function.

Let

$$\begin{array}{c} \frac{\partial \log[B\left(y_{t};\mu_{t},\phi_{t}\right)]}{\partial \mu_{t}}=\phi_{t}\left(y_{t}^{*}-\mu_{t}^{*}\right). \end{array}$$

The score function for *ω* is given by

$$\begin{array}{c} \frac{\partial \ell (\theta )}{\partial \omega_{j}}=\sum_{t=1}^{n_{0}}\frac{\partial \ell_{t}\left(\theta \right)}{\partial \alpha_{0t}}\frac{\partial \alpha_{0t}}{\partial \omega_{j}}+\sum_{t=m+1}^{n}\frac{\partial \ell_{t}\left(\theta \right)}{\partial \alpha_{0t}}\frac{\partial \alpha_{0t}}{\partial \omega_{j}}+\sum_{t=m+1}^{n}\frac{\partial \ell_{t}\left(\theta \right)}{\partial \mu_{t}}\frac{\partial \mu_{t}}{\partial \alpha_{0t}}\frac{\partial \alpha_{0t}}{\partial \omega_{j}}, \end{array}$$

where $\partial \alpha_{0t}/\partial \omega_{j}={\tilde{x}}_{tj}/g_{1}^{\prime }\left(\alpha_{0t}\right)$. Therefore,

$$\begin{array}{cc} \frac{\partial \ell (\theta )}{\partial \omega_{j}} & =\sum_{t=1}^{n}\{\frac{1}{\alpha_{0t}g_{1}^{\prime }\left(\alpha_{0t}\right)}{\tilde{x}}_{tj}1_{0}\left(y_{t}\right)-[\frac{1-\gamma_{t}}{c_{t}g_{1}^{\prime }\left(\alpha_{0t}\right)}{\tilde{x}}_{tj}\\ & -\phi_{t}\frac{\gamma_{t}\left(1-\gamma_{t}\right)\left(1-\alpha_{1t}\right)\left(y_{t}^{*}-\mu_{t}^{*}\right)}{c_{t}^{2}g_{1}^{\prime }\left(\alpha_{0t}\right)}{\tilde{x}}_{tj}]\left(1-1_{0}\left(y_{t}\right)\right)\left(1-1_{1}\left(y_{t}\right)\right)\}. \end{array}$$

The score function for *κ* is given by

$$\begin{array}{c} \frac{\partial \ell (\theta )}{\partial \kappa_{j}}=\sum_{t=n_{0}+1}^{m}\frac{\partial \ell_{t}\left(\theta \right)}{\partial \alpha_{1t}}\frac{\partial \alpha_{1t}}{\partial \kappa_{j}}+\sum_{t=m+1}^{n}\frac{\partial \ell_{t}\left(\theta \right)}{\partial \alpha_{1t}}\frac{\partial \alpha_{1t}}{\partial \kappa_{j}}+\sum_{t=m+1}^{n}\frac{\partial \ell_{t}\left(\theta \right)}{\partial \mu_{t}}\frac{\partial \mu_{t}}{\partial \alpha_{1t}}\frac{\partial \alpha_{1t}}{\partial \kappa_{j}}, \end{array}$$

where $\partial \alpha_{1t}/\partial \kappa_{j}={\overset{ˇ}{x}}_{tj}/g_{2}^{\prime }\left(\alpha_{1t}\right)$. Thus,

$$\begin{array}{cc} \frac{\partial \ell (\theta )}{\partial \kappa_{j}} & =\sum_{t=1}^{n}\{\frac{1}{\alpha_{1t}g_{2}^{\prime }\left(\alpha_{1t}\right)}{\overset{ˇ}{x}}_{tj}1_{1}\left(y_{t}\right)-[\frac{\gamma_{t}}{c_{t}g_{2}^{\prime }\left(\alpha_{1t}\right)}{\overset{ˇ}{x}}_{tj}\\ & +\phi_{t}\frac{\gamma_{t}\left(1-\gamma_{t}\right)\left(1-\alpha_{0t}\right)\left(y_{t}^{*}-\mu_{t}^{*}\right)}{c_{t}^{2}g_{2}^{\prime }\left(\alpha_{1t}\right)}{\overset{ˇ}{x}}_{tj}]\left(1-1_{0}\left(y_{t}\right)\right)\left(1-1_{1}\left(y_{t}\right)\right)\}. \end{array}$$

For *β*, the score function is given by

$$\begin{array}{c} \frac{\partial \ell (\theta )}{\partial \beta_{j}}=\sum_{t=1}^{n_{0}}\frac{\partial \ell_{t}\left(\theta \right)}{\partial \gamma_{t}}\frac{\partial \gamma_{t}}{\partial \beta_{j}}+\sum_{t=n_{0}+1}^{m}\frac{\partial \ell_{t}\left(\theta \right)}{\partial \gamma_{t}}\frac{\partial \gamma_{t}}{\partial \beta_{j}}+\sum_{t=m+1}^{n}\frac{\partial \ell_{t}\left(\theta \right)}{\partial \gamma_{t}}\frac{\partial \gamma_{t}}{\partial \beta_{j}}+\sum_{t=1}^{n}\frac{\partial \ell_{t}\left(\theta \right)}{\partial \mu_{t}}\frac{\partial \mu_{t}}{\partial \gamma_{t}}\frac{\partial \gamma_{t}}{\partial \beta_{j}}, \end{array}$$

where $\partial \gamma_{t}/\partial \beta_{j}=x_{tj}/g_{3}^{\prime }\left(\gamma_{t}\right)$ and $\partial \mu_{t}/\partial \beta_{j}=\frac{\left(1-\alpha_{0t}\right)\left(1-\alpha_{1t}\right)}{c_{t}^{2}g_{3}^{\prime }\left(\gamma_{t}\right)}x_{tj}$. Then we have

$$\begin{array}{cc} \frac{\partial \ell (\theta )}{\partial \beta_{j}} & =\sum_{t=1}^{n}\{-\frac{1}{\left(1-\gamma_{t}\right)g_{3}^{\prime }\left(\gamma_{t}\right)}x_{tj}1_{0}\left(y_{t}\right)+\frac{1}{\gamma_{t}g_{3}^{\prime }\left(\gamma_{t}\right)}x_{tj}1_{1}\left(y_{t}\right)\\ & +[\frac{\left(\alpha_{0t}-\alpha_{1t}\right)}{c_{t}g_{3}^{\prime }\left(\gamma_{t}\right)}x_{tj}+\phi_{t}\frac{\left(1-\alpha_{0t}\right)\left(1-\alpha_{1t}\right)\left(y_{t}^{*}-\mu_{t}^{*}\right)}{c_{t}^{2}g_{3}^{\prime }\left(\gamma_{t}\right)}x_{tj}]\left(1-1_{0}\left(y_{t}\right)\right)\left(1-1_{1}\left(y_{t}\right)\right)\}. \end{array}$$

The score function for *ζ* is given by

$$\begin{array}{c} \frac{\partial \ell (\theta )}{\partial \zeta_{j}}=\sum_{t=1}^{n}\frac{\partial \ell_{t}\left(\theta \right)}{\partial \phi_{t}}\frac{\partial \phi_{t}}{\partial \zeta_{j}}, \end{array}$$

where $\partial \phi_{t}/\partial \zeta_{j}={\ddot{x}}_{tj}/g_{4}^{\prime }\left(\phi_{t}\right)$. Therefore,

$$\begin{array}{c} \frac{\partial \ell (\theta )}{\partial \zeta_{j}}=\sum_{t=1}^{n}[\frac{\mu_{t}\left(y_{t}^{*}-\mu_{t}^{*}\right)+\left(y_{t}^{†}-\mu_{t}^{†}\right)}{g_{4}^{\prime }\left(\phi_{t}\right)}{\ddot{x}}_{tj}]\left(1-1_{0}\left(y_{t}\right)\right)\left(1-1_{1}\left(y_{t}\right)\right). \end{array}$$

In matrix form, each term of the score vector is given by

$$\begin{array}{cc} U_{\omega }\left(\theta \right) & ={\tilde{X}}^{⊤}T_{1}\left\{J_{1}I_{0}-\left[G_{2}-RA_{2}\left(Y^{*}-M^{*}\right)\right]I_{2}\right\},\\ U_{\kappa }\left(\theta \right) & ={\overset{ˇ}{X}}^{⊤}T_{2}\left\{J_{2}I_{1}-\left[G_{1}+RA_{1}\left(Y^{*}-M^{*}\right)\right]I_{2}\right\},\\ U_{\beta }\left(\theta \right) & =X^{⊤}T_{3}\left\{-B_{2}I_{0}+B_{1}I_{1}+\left[S_{3}+W\left(Y^{*}-M^{*}\right)\right]I_{2}\right\},\\ U_{\zeta }\left(\theta \right) & ={\ddot{X}}^{⊤}T_{4}MI_{2}, \end{array}$$

where $T_{1}=\text{diag}\{\frac{1}{g_{1}^{\prime }\left(\alpha_{01}\right)},\ldots ,\frac{1}{g_{1}^{\prime }\left(\alpha_{0n}\right)}\}$, $T_{2}=\text{diag}\{\frac{1}{g_{2}^{\prime }\left(\alpha_{11}\right)},\ldots ,\frac{1}{g_{2}^{\prime }\left(\alpha_{1n}\right)}\}$, $T_{3}=\text{diag}\{\frac{1}{g_{3}^{\prime }\left(\gamma_{1}\right)},\ldots ,\frac{1}{g_{3}^{\prime }\left(\gamma_{n}\right)}\}$, $T_{4}=\text{diag}\{\frac{1}{g_{4}^{\prime }\left(\phi_{1}\right)},\ldots ,\frac{1}{g_{4}^{\prime }\left(\phi_{n}\right)}\}$, $Y^{*}=\text{diag}\left(y_{1}^{*},\ldots ,y_{n}^{*}\right)$, $M^{*}=\text{diag}\left(\mu_{1}^{*},\ldots ,\mu_{n}^{*}\right)$, *M* = diag{*m*_1_, …, *m*_*n*_}, $m_{t}=\mu_{t}\left(y_{t}^{*}-\mu_{t}^{*}\right)+\left(y_{t}^{†}-\mu_{t}^{†}\right)$, *A*_1_ = diag{(1 − *α*_01_), …, (1 − *α*_0*n*_)}, *A*_2_ = diag{(1 − *α*_11_), …, (1 − *α*_1*n*_)}, $B_{1}=\text{diag}\{\frac{1}{\gamma_{1}},\ldots ,\frac{1}{\gamma_{n}}\}$, $B_{2}=\text{diag}\{\frac{1}{1-\gamma_{1}},\ldots ,\frac{1}{1-\gamma_{n}}\}$, $R=\text{diag}\{\frac{\phi_{1}\gamma_{1}\left(1-\gamma_{1}\right)}{c_{1}^{2}},\ldots ,\frac{\phi_{n}\gamma_{n}\left(1-\gamma_{n}\right)}{c_{n}^{2}}\}$, $J_{1}=\text{diag}\{\frac{1}{\alpha_{01}},\ldots ,\frac{1}{\alpha_{0n}}\}$, $J_{2}=\text{diag}\{\frac{1}{\alpha_{11}},\ldots ,\frac{1}{\alpha_{1n}}\}$, $W=\text{diag}\{\frac{\phi_{1}\left(1-\alpha_{01}\right)\left(1-\alpha_{11}\right)}{c_{1}^{2}},\ldots ,\frac{\phi_{n}\left(1-\alpha_{0n}\right)\left(1-\alpha_{1n}\right)}{c_{n}^{2}}\}$, $G_{1}=\text{diag}\{\frac{\gamma_{1}}{c_{1}},\ldots ,\frac{\gamma_{n}}{c_{n}}\}$, $G_{2}=\text{diag}\{\frac{1-\gamma_{1}}{c_{1}},\ldots ,\frac{1-\gamma_{n}}{c_{n}}\}$, $S_{3}=\text{diag}\{\frac{\alpha_{01}-\alpha_{11}}{c_{1}},\ldots ,\frac{\alpha_{0n}-\alpha_{1n}}{c_{n}}\}$, $\tilde{X}$ is a *n* × *k*_1_ matrix whose *t*-th row is ${\tilde{x}}_{t}^{⊤}$, $\overset{ˇ}{X}$ is a *n* × *k*_2_ matrix whose *t*-th row is ${\overset{ˇ}{x}}_{t}^{⊤}$, *X* is a *n* × *k*_3_ matrix whose *t*-th row is $x_{t}^{⊤}$, $\ddot{X}$ is a *n* × *k*_4_ matrix whose *t*-th row is ${\ddot{x}}_{t}^{⊤}$, $I_{0}={\left(1_{0}\left(y_{1}\right),\ldots ,1_{0}\left(y_{n}\right)\right)}^{⊤}$, $I_{1}={\left(1_{1}\left(y_{1}\right),\ldots ,1_{1}\left(y_{n}\right)\right)}^{⊤}$, and $I_{2}={\left(\left(1-1_{0}\left(y_{1}\right)\right)\left(1-1_{1}\left(y_{1}\right)\right),\ldots ,\left(1-1_{0}\left(y_{n}\right)\right)\left(1-1_{1}\left(y_{n}\right)\right)\right)}^{⊤}$.

The joint information matrix for the parameter vector ***θ*** = (*ω*^⊤^, *κ*^⊤^, *β*^⊤^, *ζ*^⊤^)^⊤^ is given by

$$\begin{array}{c} K\left(\theta \right)=(\begin{array}{cccc} K_{\omega \omega } & K_{\omega \kappa } & K_{\omega \beta } & K_{\omega \zeta }\\ K_{\kappa \omega } & K_{\kappa \kappa } & K_{\kappa \beta } & K_{\kappa \zeta }\\ K_{\beta \omega } & K_{\beta \kappa } & K_{\beta \beta } & K_{\beta \zeta }\\ K_{\zeta \omega } & K_{\zeta \kappa } & K_{\zeta \beta } & K_{\zeta \zeta } \end{array}), \end{array}$$

where $K_{\omega \omega }={\tilde{X}}^{⊤}T_{1}^{2}\{\frac{J_{1}}{B_{2}}+\left[G_{2}^{2}+R^{2}A_{2}^{2}V^{*}\right]C\}\tilde{X}$, $K_{\omega \kappa }=K_{\kappa \omega }^{⊤}={\tilde{X}}^{⊤}T_{1}T_{2}\left[G_{1}G_{2}-R^{2}A_{1}A_{2}V^{*}\right]C\overset{ˇ}{X}$, $K_{\omega \beta }=K_{\beta \omega }^{⊤}={\tilde{X}}^{⊤}T_{1}T_{3}\left[-S_{2}+WA_{2}RV^{*}\right]CX$, $K_{\omega \zeta }=K_{\zeta \omega }^{⊤}={\tilde{X}}^{⊤}T_{1}T_{4}RA_{2}HC\ddot{X}$, $K_{\kappa \kappa }={\overset{ˇ}{X}}^{⊤}T_{2}^{2}\{\frac{J_{2}}{B_{1}}+$ $+\left[G_{1}^{2}+R^{2}A_{1}^{2}V^{*}\right]C\}\overset{ˇ}{X}$, $K_{\kappa \beta }=K_{\beta \kappa }^{⊤}={\overset{ˇ}{X}}^{⊤}T_{2}T_{3}\left[S_{1}-WA_{1}RV^{*}\right]CX$, $K_{\kappa \zeta }=K_{\zeta \kappa }^{⊤}=-{\overset{ˇ}{X}}^{⊤}T_{2}T_{4}RA_{1}HC\ddot{X}$, $K_{\beta \beta }=X^{⊤}T_{3}^{2}\{\frac{B_{2}}{J_{1}}+\frac{B_{1}}{J_{2}}+\left[S_{3}^{2}+W^{2}V^{*}\right]C\}X$, $K_{\beta \zeta }=K_{\zeta \beta }^{⊤}=X^{⊤}T_{3}T_{4}WHC\ddot{X}$, $K_{\zeta \zeta }={\ddot{X}}^{⊤}T_{4}^{2}DC\ddot{X}$, $S_{1}=\text{diag}\{\frac{1-\alpha_{01}}{c_{1}^{2}},\ldots ,\frac{1-\alpha_{0n}}{c_{n}^{2}}\}$, $S_{2}=\text{diag}\{\frac{1-\alpha_{11}}{c_{1}^{2}},\ldots ,\frac{1-\alpha_{1n}}{c_{n}^{2}}\}$, $V^{*}=\text{diag}\{v_{1}^{*},\ldots ,v_{n}^{*}\}$, $v_{t}^{*}=\psi^{\prime }\left(\mu_{t}\phi_{t}\right)+\psi^{\prime }\left(\left(1-\mu_{t}\right)\phi_{t}\right)$, $H=\text{diag}\{\mu_{1}v_{1}^{*}+f_{1},\ldots ,\mu_{n}v_{n}^{*}+f_{n}\}$, *f*_*t*_ = −*ψ*′((1 − *μ*_*t*_)*ϕ*_*t*_), *D* = diag{*d*_1_, …, *d*_*n*_}, $d_{t}=\psi^{\prime }\left(\phi_{t}\right)-\psi^{\prime }\left(\mu_{t}\phi_{t}\right)\mu_{t}^{2}-\psi^{\prime }\left(\left(1-\mu_{t}\right)\phi_{t}\right){\left(1-\mu_{t}\right)}^{2}$, *C* = diag{*c*_1_, …, *c*_*n*_}, and *c*_*t*_ = 1 − *α*_0*t*_(1 − *γ*_*t*_) − *α*_1*t*_ *γ*_*t*_.

## Supporting information

S1 Dataset (TXT)
